# Supplementary material for: The interactome of intact mitochondria by cross-linking mass spectrometry provides evidence for coexisting respiratory supercomplexes
Source: Mol Cell Proteomics. 2017 Dec 8;17(2):216–32. doi: 10.1074/mcp.RA117.000470 (PMC5795388; doi:10.1074/mcp.RA117.000470)
Supplement: Supplemental Data [file supp_RA117.000470_133922_0_supp_23978_fzffwf.zip › spectra_annotation/mito_spectra_annotation/364-1-5-1-88-1.pdf]

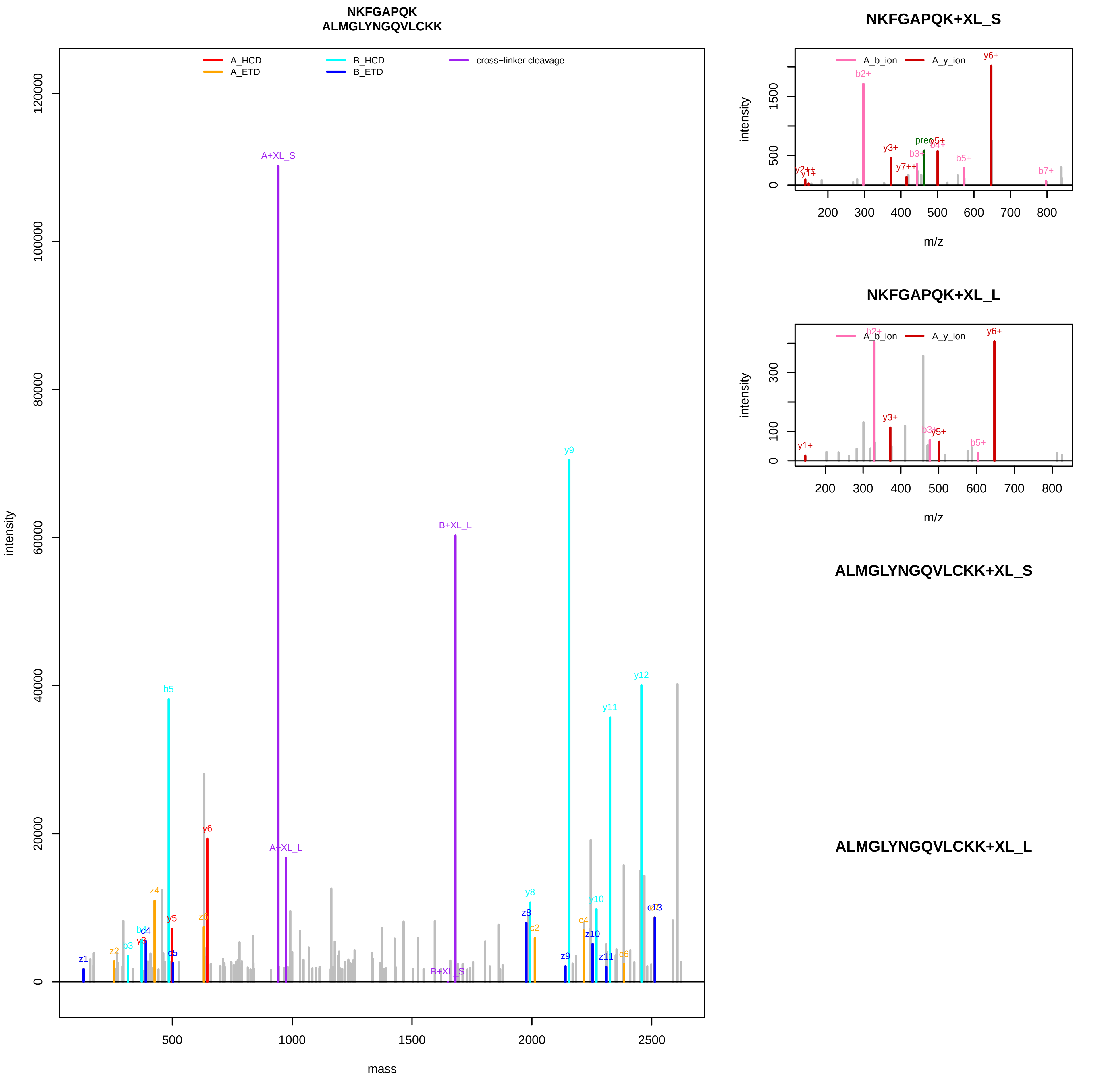
NKFGAPQK
ALMGLYNGQVLCKK
A_HCD
A_ETD
B_HCD
B_ETD
cross−linker cleavage
A+XL_S
B+XL_L
A+XL_L
B+XL_S
y9
y12
y11
y10
y8
z8
c2
c4
z10
z9
z11
c6
c13
b5
y6
y5
z6
z4
z2
z1
b3
c4
y3
c5
intensity
mass
0
20000
40000
60000
80000
100000
120000
500
1000
1500
2000
2500
NKFGAPQK+XL_S
A_b_ion
A_y_ion
y6+
b2+
y3+
prec
y5+
b4+
b3+
y7++
b5+
b7+
y2++
y1+
0
500
1500
200
300
400
500
600
700
800
m/z
NKFGAPQK+XL_L
b2+
y3+
b3+
y5+
b5+
y1+
0
100
300
ALMGLYNGQVLCKK+XL_S
ALMGLYNGQVLCKK+XL_L
